# The Other Syndemic: HIV and Obesity

**DOI:** 10.1007/s11695-024-07444-6

**Published:** 2024-09-16

**Authors:** Francis M. Finucane

**Affiliations:** 1 College of Medicine, Nursing and Health Sciences, University of Galway, Galway, Ireland; 2 CÚRAM, University of Galway, Galway, Ireland

**Keywords:** Obesity, HIV, AIDS, Syndemic, Bariatric surgery

As epidemics go, HIV/ AIDS and obesity have much in common. Both are major public health challenges of our time, with HIV/AIDS being the third leading cause of morbidity and mortality from communicable diseases globally in 2019 [1]. Like obesity, the drivers of the HIV/ AIDS epidemic are largely societal and structural and aggravated by bias and discrimination against affected individuals [2]. As effective treatments for obesity have become a reality, so too has HIV infection, previously considered a terminal illness inevitably manifesting as AIDS, become a chronic condition requiring long-term clinical management, thanks to the development of effective antiretroviral therapy (ART) [3]. Unfortunately, ART is known to cause weight gain and has contributed to a higher incidence of obesity and related disorders in people living with HIV [4].

In this issue of Obesity Surgery, Fehervar and colleagues describe a retrospective cohort study of changes in weight over five years in ten patients with HIV who underwent treatment for severe obesity with bariatric surgery at a single center in London, UK. As anticipated, patients lost weight after their surgery. While the authors conclude that “bariatric surgery appears to be a safe and effective medium-term treatment for obesity in patients living with HIV,” larger prospective cohort studies and randomized controlled trials would be necessary to confirm this, as safety issues may not become apparent in a small retrospective single-center study such as this. The same is true for inferences that weight loss occurred “while maintaining the efficacy of HIV treatments.” On average, patients in this study who had surgery were 9 years younger, 26 kg heavier, smoked more, and had a 43% higher CD4 count than otherwise similar patients at the same center who underwent a conventional lifestyle modification program to treat their obesity, with no statistically significant change in weight. As information about specific HIV treatments in the two groups is not provided in the paper, and they were not randomized to surgery versus lifestyle alone treatment, we will have to wait and see what adequately designed and powered studies tell us about the influence of obesity surgery on HIV treatment efficacy and safety.

These studies cannot come soon enough. The latest generation of ARTs, such as the integrase inhibitor dolutegravir are more inclined to cause weight gain than older drugs [5]. In the ADVANCE trial of HIV treatment, women on an ART regimen that included a tenofovir alafenamide fumarate (TAF) “backbone” gained an average of 10 kg over 96 weeks [6]. The mechanisms underlying the development of obesity and related disorders in people taking ART for HIV are poorly understood. Still, they may include less nausea, more sleep disturbance, and more lipohypertrophy with integrase inhibitors compared to (older) protease inhibitors [5]. Dolutegravir binds to and inhibits the human melanocortin-4 receptor, which might be expected to increase appetite, though in vitro studies suggest this is not its primary influence on body weight control [7]. While ART has a negative impact on body weight, the converse is also true: Obesity is known to affect HIV drug bioavailability and treatment efficacy adversely [8].

Fehervar and colleagues are to be commended for highlighting an important, if neglected, area of bariatric clinical practice. The largest previous study of obesity surgery and HIV had just 58 patients [9]. Most are much smaller, with limited data on important clinical outcomes [10]. We must do better in light of these preliminary, exploratory findings. Recently published expert consensus on the metabolic and cardiovascular risks faced by people living with HIV receiving ART [11] did not mention the need for a better understanding of the role of bariatric surgery or its influence on HIV outcomes in those patients also affected by severe obesity. Meanwhile, randomized controlled trials of drug treatment for obesity in patients living with HIV have begun in earnest and are already showing benefits [12]. Investigating the role of obesity surgery in patients living with HIV requires urgent and immediate clinical research prioritization and funding.
